# Triglyceride-glucose index and triglyceride to high-density lipoprotein cholesterol ratio as potential cardiovascular disease risk factors: an analysis of UK biobank data

**DOI:** 10.1186/s12933-023-01762-2

**Published:** 2023-02-16

**Authors:** Bizhong Che, Chongke Zhong, Ruijie Zhang, Liyuan Pu, Tian Zhao, Yonghong Zhang, Liyuan Han

**Affiliations:** 1 Department of Global Health, Ningbo Institute of Life and Health Industry, University of Chinese Academy of Sciences, 159 Beijiao Road, Jiangbei District, Ningbo, 153000 Zhejiang China; 2 Department of Epidemiology, School of Public Health and Jiangsu Key Laboratory of Preventive and Translational Medicine for Geriatric Diseases, Suzhou Medical College of Soochow University, 199 Renai Road, Industrial Park District, Suzhou, 215123 Jiangsu China; 3 Hwa Mei Hospital, Key Laboratory of Diagnosis and Treatment of Digestive System Tumors of Zhejiang Province, University of Chinese Academy of Sciences, Ningbo, Zhejiang China

**Keywords:** Cardiovascular disease, Insulin resistance, TyG index, TG/HDL‑C ratio, Mediation analysis

## Abstract

**Background:**

The triglyceride-glucose (TyG) index and triglyceride to high-density lipoprotein cholesterol (TG/HDL-C) ratio, two simple surrogate indicators of insulin resistance, have been demonstrated to predict cardiovascular disease (CVD). However, very few studies have investigated their associations with CVD in European populations.

**Methods:**

A total of 403,335 participants from the UK Biobank with data for TyG index and TG/HDL-C ratio and free from CVD at baseline were included. Cox models were applied to evaluate the association between TyG index and TG/HDL-C ratio and incident CVD. Mediation analyses were performed to evaluate the contribution of prevalent diabetes, hypertension, and dyslipidemia to observed associations.

**Results:**

During a median follow-up of 8.1 years, 19,754 (4.9%) individuals developed CVD, including 16,404 (4.1%) cases of CHD and 3976 (1.0%) cases of stroke. The multivariable-adjusted hazard ratios of total CVD in higher quartiles versus the lowest quartiles were 1.05, 1.05, and 1.19, respectively, for TyG index, and 1.07, 1.13, and 1.29, respectively, for TG/HDL-C ratio. There were significant trends toward an increasing risk of CVD across the quartiles of TyG index and TG/HDL-C ratio. In mediation analyses, dyslipidemia, type 2 diabetes, and hypertension explained 45.8%, 27.0%, and 15.0% of TyG index’s association with CVD, respectively, and 40.0%, 11.8%, and 13.3% of TG/HDL-C ratio’s association with CVD, respectively.

**Conclusions:**

Elevated baseline TyG index and TG/HDL-C ratio were associated with a higher risk of CVD after adjustment for the well-established CVD risk factors. These associations were largely mediated by greater prevalence of dyslipidemia, type 2 diabetes, and hypertension.

**Supplementary Information:**

The online version contains supplementary material available at 10.1186/s12933-023-01762-2.

## Introduction

Cardiovascular disease (CVD), including ischemic heart disease and stroke, constitute the leading cause of premature death worldwide [1]. In 2017, CVD caused an estimated 17.8 million deaths and was responsible for 330 million years of life lost globally [1]. This highlights the importance of identifying risk factors that could predict the risk of CVD and thereby facilitate its prevention at an early stage.

Insulin resistance, a pathophysiological condition characterized by the decreased insulin sensitivity of peripheral tissues, plays a key role in the development of metabolic syndrome and atherosclerosis [2, 3]. The euglycemic-hyperinsulinemic clamp is served as the gold standard to identify insulin resistance, but the technique is laborious, costly, and therefore impractical in the clinical setting [4]. The triglyceride-glucose (TyG) index and triglyceride to high-density lipoprotein cholesterol (TG/HDL-C) ratio have been proposed as simple and credible surrogate indicators of insulin resistance because they show strong correlations with the euglycemic-hyperinsulinemic clamp and they are suitable for clinical practice and large epidemiological studies [5, 6]. Several cross-sectional and retrospective studies have reported significant associations of the TyG index and TG/HDL-C ratio with incident CVD [7–12]. Most prospective cohort studies on the predictive value of the TyG index and TG/HDL-C ratio for CVD risk have been conducted in Asian populations [13–18], and few in European and American populations [19–21].

The pathophysiologic mechanisms known to increase CVD risk in individuals with insulin resistance include diabetes, hypertension, and dyslipidemia [22, 23], suggesting that the effect of the TyG index and TG/HDL-C ratio on CVD might be partly mediated through these comorbidities. However, to the best of our knowledge, no study to date has explored the mediating role of prevalent diabetes, hypertension, and dyslipidemia in the associations of the TyG index and TG/HDL-C ratio with CVD risk.

Using data from the UK Biobank, we aimed 1) to comprehensively investigate the associations of the TyG index and TG/HDL-C ratio with the risk of CVD, and 2) to quantify the contribution of prevalent diabetes, hypertension, and dyslipidemia as potential mediators in the effect of the TyG index and TG/HDL-C ratio on the risk of CVD.

## Methods

### Study design and participants

UK Biobank is a community-based prospective cohort of over half a million individuals aged 40–69 years at recruitment between 2006 and 2010. Potential participants attended one of 22 assessment centers across England, Wales, and Scotland where they underwent physical examinations, provided biological samples, and completed baseline questionnaires, as described in detail elsewhere [24]. After excluding those who had missing data on the TyG index or TG/HDL-C ratio (n = 73,128), withdrew at the time of the study (n = 1298), or had prevalent CVD at baseline (n = 24,744), a total of 403,335 individuals remained for the final analysis.

UK Biobank was constructed under ethical approval obtained by the North West Multi-Centre Research Ethics Committee (REC reference: 11/NW/03820) and all participants provided written informed consent prior to participation. The current analyses were carried out under Application Number 52632.

### Data collection

We used the baseline touchscreen questionnaire to derive information on several potential confounders: age, sex, ethnicity, Townsend Deprivation Index, current smoking status, physical activity (< 150 or  ≥ 150 min/week based on the total time spent in moderate physical activity in minutes each week [25]), and medication use at baseline (aspirin, insulin, and antihypertensive and cholesterol-lowering medications). The Townsend deprivation index is a composite measure of deprivation based on unemployment, non-car ownership, non-home ownership, and household overcrowding. It is derived from the residential postcode, with a negative value representing high socioeconomic status [26]. Two measurements of systolic and diastolic blood pressure were taken using the Omron HEM-7015IT digital blood pressure monitor or a manual sphygmomanometer, and the mean of the two measurements was used for analysis. Body mass index was calculated as the weight in kilograms (kg) divided by the square of the height in meters (m^2^).

Peripheral venous blood samples were collected at baseline from all participants, and collection procedures for the UK Biobank study were validated [27]. Blood samples were taken at random due to their future applicability to a wide range of diseases and the difficulty in collecting and processing fasting blood samples in a very large population with a distributed assessment center setting [27]. Non-fasting serum biochemical measurements including glucose, triglycerides (TG), total cholesterol, high density lipoprotein cholesterol (HDL-C), low density lipoprotein cholesterol (LDL-C), high sensitivity C-reactive protein, creatinine, and uric acid were performed on a Beckman Coulter AU5800 clinical chemistry analyzer at the central laboratory. Coefficients of variation for concentrations of TG, high sensitivity C-reactive protein, and creatinine were less than 3% and for glucose, total cholesterol, HDL-C, LDL-C, and uric acid were less than 2%. Glycated hemoglobin (HbA_1c_) was measured by high-performance liquid chromatography analysis on a Bio-Rad VARIANT II Turbo. The TyG index was calculated using the formula: ln [triglycerides (mg/dL) × glucose (mg/dL)/2] [28]. The TG/HDL-C ratio was calculated as TG (mg/dL) divided by HDL-C (mg/dL). Estimated glomerular filtration rate (eGFR) was calculated using the Chronic Kidney Disease Epidemiology Collaboration creatinine equation [29], based on the measurement of creatinine at baseline visit. Chronic kidney disease was defined as an eGFR < 60 mL/min/1.73 m^2^ [30]. Details of these assessments can be found in the UK Biobank’s online protocol (www.ukbiobank.ac.uk).

### Outcomes and comorbidities

The primary outcome of this study was incident CVD, defined as fatal or non-fatal coronary heart disease (CHD) or stroke. The secondary outcomes were individual diagnoses of CHD and stroke. Participant follow-up started at inclusion in the UK Biobank and was censored on March 31, 2017 or on the date of the first CHD or stroke. The date of the first incident CHD or stroke after baseline was ascertained through record linkage with hospital episode statistics in England, Scotland, and Wales and national death registers. Incident CHD was defined by the 10th revision of the International Classification of Diseases (ICD-10) codes I20–I25. Incident stroke was defined by ICD codes I60–I64. Prevalent comorbidities including dyslipidemia (ICD-10 codes E78), type 1 diabetes (ICD-10 code E10), type 2 diabetes (ICD-10 code E11), retinopathy (ICD-10 codes E10.3, E11.3, E12.3, E13.3, E14.3, H28.0, H33, H35.3, H36.0, H40–H42) and hypertension (ICD-10 codes I10–I15) were captured from the health records (Additional file 1: Appendix S1).

### Statistical analyses

The data were expressed as mean (SD) for continuous variables and frequency (percentage) for categorical variables. Participants were stratified into four groups according to the quartiles of the TyG index and TG/HDL-C ratio at baseline. Trends across quartiles were tested by the generalized linear regression analysis for continuous variables and the Cochran-Armitage trend chi-square test for categorical variables, respectively. Pearson’s (for continuous variables) and Point-biserial (for dichotomized variables) correlation tests were used to assess the correlations of the TyG index and TG/HDL-C ratio with participant characteristics.

Kaplan-Meier cumulative incidence plots were generated to assess the relationship between the quartiles of the TyG index and TG/HDL-C ratio and incident CVD (including total CVD, CHD, and stroke) during follow-up, and the log-rank test was used for statistical assessment. Cox proportional hazard models were applied to calculate hazard ratios and 95% confidence intervals of higher quartiles in relation to the lowest quartiles and of 1-SD increments in log-transformed TyG index and TG/HDL-C ratio for all endpoints. *P* values for linear trends in hazard ratios across the quartiles of the TyG index and TG/HDL-C ratio were tested using the median value within each quartile as the predictor. Three models were established with incremental degrees of adjustment for potential confounders of CVD: model 1 was adjusted for none, model 2 was adjusted for baseline age (years), sex, ethnicity (White and others), region (England, Scotland, and Wales), and Townsend Deprivation Index, and model 3 was adjusted for the same variables as model 2 and also for current smoking status (yes or no), physical activity (< 150 or  ≥ 150 min/week), body mass index (kg/m^2^), systolic blood pressure (mm Hg), total cholesterol (mg/dL), LDL-C (mg/dL), uric acid (mg/dL), HbA_1c_ (mmol/mol), eGFR (mL/min/1.73 m^2^), high sensitivity C-reactive protein (mg/L), aspirin use (yes or no), undergoing insulin treatment (yes or no), use of antihypertensive medication (yes or no), use of cholesterol-lowering medication (yes or no), prevalent retinopathy (yes or no), and chronic kidney disease (yes or no). To determine whether there was a nonlinear dose-response relationship of the TyG index and TG/HDL-C ratio with the risk of CVD after multivariable-adjustment, restricted cubic splines were fitted, with four knots placed at the 5th, 35th, 65th, and 95th percentiles and the 1% highest and lowest TyG index and TG/HDL-C ratio observations were trimmed.

Mediation analysis was performed using the publicly available SAS macro %mediate (http://www.hsph.harvard.edu/donna-spiegelman/software/mediate/) [31] to evaluate the proportional contribution of prevalent dyslipidemia, diabetes, and hypertension on the associations of the TyG index and TG/HDL-C ratio with CVD risk. In brief, the mediation proportion is a statistical measure used to estimate how much of the total exposure-outcome association is explained by a particular mediator [32]. Mediation analysis models were adjusted for the same set of confounders as in model 3 of the primary analyses. In sensitivity analyses, we first adjusted for prevalent dyslipidemia, diabetes, and hypertension in addition to the variables in model 3. Moreover, to check for reverse causation, we excluded individuals who developed CVD within the first 3 years of follow-up.

The proportion of missing data on covariates ranged from 0.01% to 19.1%, and multiple imputation with the Markov chain Monte Carlo method was performed to assign any missing covariate data. Statistical significance was determined as a two-sided *P* value less than 0.05. Statistical analysis was conducted using SAS statistical software version 9.4 (SAS Institute, Cary, NC, USA).

## Results

### Participant characteristics

Participant characteristics both overall and stratified by the quartiles of the TyG index were presented in Table 1, and for the TG/HDL-C ratio in Additional file 1: Table S1. Of 403,335 included participants, 222,586 (55.2%) were women, 381,880 (94.7%) were White, and the mean (SD) age at baseline was 56.2 (8.1) years. The prevalence of dyslipidemia, type 1 diabetes, type 2 diabetes, and hypertension were 6.7%, 0.3%, 3.5%, and 13.9%, respectively. The median TyG index and TG/HDL-C ratio were 8.67 (IQR 8.31–9.07) and 2.39 (IQR 1.49–3.90), respectively. Higher TyG index and TG/HDL-C ratio were both associated with male sex, older age, White ethnicity, lower socioeconomic status, smoking, less physical activity, higher body mass index and blood pressure, increased serum concentrations of uric acid, glycated hemoglobin, total cholesterol, LDL-C, C-reactive protein, and creatinine="null">, lower eGFR, and more frequent use of aspirin, insulin, and antihypertensive and cholesterol-lowering medication at baseline. Moreover, individuals with higher TyG index or TG/HDL-C ratio levels had greater prevalence of retinopathy="null">, chronic kidney disease, dyslipidemia, type 1 diabetes, type 2 diabetes, and hypertension than those with lower levels (all *P* < 0.001 for trend). The results of Pearson’s or Point-biserial correlations of the TyG index and TG/HDL-C ratio with participant characteristics were displayed in Additional file 1: Table S2. Significant differences in participant characteristics were observed in England, Scotland, and Wales (*P* < 0.001 for difference), except for triglycerides, the TG/HDL-C ratio, and insulin use (Additional file 1: Table S3).

**Table 1:** Characteristics of the study population according to the TyG index quartiles

Characteristics^a^	TyG index					*P* trend
	Total	Quartile 1 < 8.31	Quartile 2 8.31–8.66	Quartile 3 8.67–9.06	Quartile 4 ≥ 9.07	
N	403,335	100,844	100,869	100,870	100,752	
Female	222,586 (55.2)	68,827 (68.3)	60,325 (59.8)	52,164 (51.7)	41,270 (41.0)	< 0.001
Age, years	56.2 (8.1)	54.0 (8.2)	56.5 (8.0)	57.3 (7.8)	57.2 (7.8)	< 0.001
White ethnicity	381,880 (94.7)	94,063 (93.3)	95,877 (95.0)	96,200 (95.4)	95,740 (95.0)	< 0.001
Townsend Deprivation Index	− 1.3 (3.1)	− 1.4 (3.1)	− 1.4 (3.0)	− 1.4 (3.0)	− 1.2 (3.1)	< 0.001
Current smoking	42,025 (10.4)	8644 (8.6)	9782 (9.7)	10,702 (10.6)	12,897 (12.8)	< 0.001
Physical activity, min/week						< 0.001
< 150	184,414 (45.7)	42,120 (41.8)	44,954 (44.6)	47,028 (46.6)	50,312 (49.9)	
≥ 150	218,921 (54.3)	58,724 (58.2)	55,915 (55.4)	53,842 (53.4)	50,440 (50.1)	
Body mass index, kg/m^2^	27.3 (4.7)	25.1 (4.0)	26.7 (4.5)	28.0 (4.6)	29.4 (4.7)	< 0.001
Systolic blood pressure, mm Hg	137.9 (18.6)	132.1 (18.4)	137.2 (18.5)	140.0 (18.2)	142.3 (17.8)	< 0.001
Diastolic blood pressure, mm Hg	82.5 (10.1)	79.5 (9.9)	82.0 (9.9)	83.5 (9.9)	84.8 (9.9)	< 0.001
Uric acid, mg/dL	5.2 (1.3)	4.6 (1.2)	5.0 (1.2)	5.3 (1.3)	5.8 (1.3)	< 0.001
Glycated hemoglobin, mmol/mol	35.9 (6.5)	34.2 (4.5)	35.1 (4.7)	35.9 (5.1)	38.4 (9.4)	< 0.001
Glucose, mg/dL	91.9 (21.3)	85.1 (10.0)	88.8 (10.9)	91.3 (13.9)	102.4 (35.3)	< 0.001
Triglycerides, mg/dL	153.9 (90.5)	73.8 (15.7)	112.0 (17.1)	158.4 (26.1)	271.4 (97.9)	< 0.001
Total cholesterol, mg/dL	222.6 (43.2)	208.6 (37.9)	220.4 (40.1)	227.5 (42.4)	234.1 (47.6)	< 0.001
High-density lipoprotein cholesterol, mg/dL	56.4 (14.7)	65.2 (15.2)	59.3 (13.9)	53.9 (12.3)	47.4 (10.9)	< 0.001
Low-density lipoprotein cholesterol, mg/dL	139.4 (33.0)	125.6 (28.0)	138.0 (30.4)	145.4 (32.6)	148.6 (35.7)	< 0.001
High sensitivity C-reactive protein, mg/L	2.6 (4.3)	1.9 (4.2)	2.4 (4.4)	2.8 (4.4)	3.1 (4.2)	< 0.001
Creatinine, mg/dL	0.81 (0.20)	0.78 (0.17)	0.80 (0.18)	0.82 (0.20)	0.84 (0.23)	< 0.001
eGFR, mL/min/1.73 m^2^	91.3 (13.2)	93.7 (12.6)	91.1 (12.7)	90.0 (13.1)	90.3 (14.0)	< 0.001
Aspirin	42,153 (10.4)	7779 (7.7)	9539 (9.5)	10,910 (10.8)	13,925 (13.8)	< 0.001
Insulin	3702 (0.9)	610 (0.6)	450 (0.4)	654 (0.7)	1988 (2.0)	< 0.001
Antihypertensive medication	73,657 (18.3)	10,886 (10.8)	16,097 (16.0)	20,496 (20.3)	26,178 (26.0)	< 0.001
Cholesterol-lowering medication	55,459 (13.7)	7907 (7.8)	11,489 (11.4)	14,804 (14.7)	21,259 (21.1)	< 0.001
Retinopathy	8868 (2.2)	1753 (1.7)	2105 (2.1)	2286 (2.3)	2724 (2.7)	< 0.001
Chronic kidney disease	7956 (2.0)	1111 (1.0)	1648 (1.6)	2263 (2.2)	2934 (2.9)	< 0.001
Dyslipidemia	27,032 (6.7)	3337 (3.3)	5414 (5.4)	7506 (7.4)	10,775 (10.7)	< 0.001
Type 1 diabetes	1275 (0.3)	184 (0.2)	163 (0.2)	222 (0.2)	706 (0.7)	< 0.001
Type 2 diabetes	14,157 (3.5)	941 (0.9)	1847 (1.8)	3262 (3.2)	8107 (8.1)	< 0.001
Hypertension	56,202 (13.9)	8595 (8.5)	12,700 (12.6)	15,657 (15.5)	19,250 (19.1)	< 0.001

TyG, triglyceride-glucose; eGFR, estimated glomerular filtration rate

^a^Continuous variables are expressed as mean (SD). Categorical variables are expressed as frequency (percentage)

### Associations of baseline TyG index and TG/HDL-C ratio with incident CVD

**Fig. 1:**
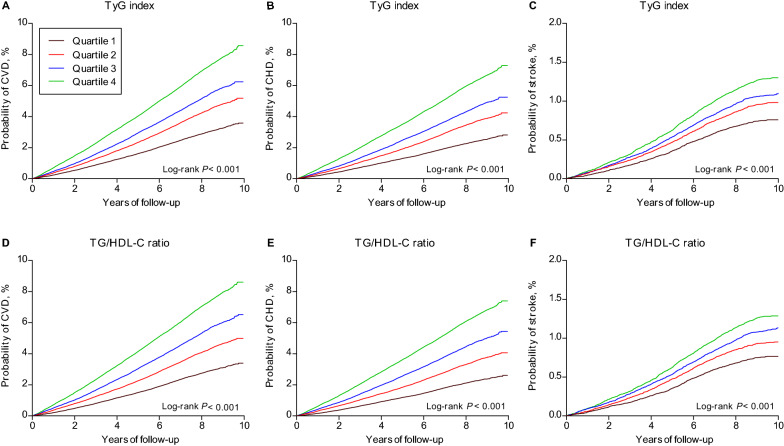
Cumulative Kaplan-Meier estimates of cardiovascular disease according to the quartiles of the TyG index **A**–**C** and TG/HDL-C ratio **D**–**F**. TyG, triglyceride-glucose; TG/HDL-C, triglyceride to high-density lipoprotein cholesterol. The quartile 1, quartile 2, quartile 3, quartile 4 were  < 8.31, 8.31–8.66, 8.67–9.06,  ≥ 9.07, respectively, for the TyG index and  < 1.49, 1.49–2.38, 2.39–3.90,  ≥ 3.90, respectively, for the TG/HDL-C ratio

During a median follow-up of 8.1 years (IQR 7.4–8.8, totaling 3,222,292 person-years), 19,754 (4.9%) participants developed CVD, including 16,404 (4.1%) cases of CHD and 3976 (1.0%) cases of stroke. There were differences in rates of incident CVD in England, Scotland, and Wales (Additional file 1: Table S3). Kaplan-Meier curves indicated a graded increased risk of CVD with higher quartiles of the TyG index and TG/HDL-C ratio (all log-rank *P* < 0.001, Fig. 1). In unadjusted Cox regression analyses, both higher TyG index and TG/HDL-C ratio were associated with increased risks of total CVD, CHD, and stroke, and these associations persisted after adjusting for sociodemographic factors. After full covariate adjustment, the hazard ratios (95% confidence intervals) of total CVD in higher quartiles versus the lowest quartiles were 1.05 (1.00–1.10), 1.05 (1.00–1.10), and 1.19 (1.14–1.25), respectively, for the TyG index, and 1.07 (1.02–1.13), 1.13 (1.07–1.18), and 1.29 (1.23–1.36), respectively, for the TG/HDL-C ratio. There were significant trends toward an increasing risk of CVD across the quartiles of the TyG index and TG/HDL-C ratio (both *P* trend  < 0.001). Each 1-SD increment in log-transformed values of the TyG index and TG/HDL-C ratio was associated with 8% and 12% increase in the risk of CVD, respectively (Tables 2 and 3). Similarly, in the fully adjusted model, hazard ratios (95% confidence intervals) of CHD in higher quartiles versus the lowest quartiles were 1.07 (1.01–1.13), 1.09 (1.03–1.15), and 1.25 (1.18–1.32), respectively, for the TyG index, and 1.11 (1.05–1.17), 1.18 (1.11–1.24), and 1.37 (1.30–1.45), respectively, for the TG/HDL-C ratio. The trends across quartiles were significant for both the TyG index and TG/HDL-C ratio (both *P* trend < 0.001). Per 1-SD increment in log-transformed values of the TyG index and TG/HDL-C ratio was associated with 10% and 15% increase in the risk of CHD, respectively. After full adjustment, the associations of the TyG index and TG/HDL-C ratio with stroke were attenuated to non-significance (Tables 2 and 3).

**Table 2:** Prospective associations between the TyG index and risk of cardiovascular diseases in the UK Biobank

	TyG index				*P* trend	Per 1 SD increase
	Quartile 1	Quartile 2	Quartile 3	Quartile 4		in Log TyG values
Median	8.07	8.49	8.86	9.36		
Cardiovascular disease						
No. of cases/person-years	2985/816,110	4351/807,104	5298/802,743	7120/796,334	< 0.001	
Model 1, HR (95% CI)	1.00	1.48 (1.41–1.55)	1.81 (1.73–1.89)	2.45 (2.35–2.56)	< 0.001	1.38 (1.36–1.40)
Model 2, HR (95% CI)	1.00	1.22 (1.17–1.28)	1.37 (1.31–1.43)	1.75 (1.68–1.83)	< 0.001	1.24 (1.22–1.26)
Model 3, HR (95% CI)	1.00	1.05 (1.00–1.10)	1.05 (1.00–1.10)	1.19 (1.14–1.25)	< 0.001	1.08 (1.06–1.10)
Coronary heart disease						
No. of cases/person-years	2338/818,425	3537/810,134	4429/806,026	6100/800,146	< 0.001	
Model 1, HR (95% CI)	1.00	1.53 (1.45–1.61)	1.93 (1.83–2.03)	2.67 (2.55–2.80)	< 0.001	1.42 (1.40–1.44)
Model 2, HR (95% CI)	1.00	1.27 (1.20–1.34)	1.46 (1.39–1.54)	1.90 (1.81–2.00)	< 0.001	1.28 (1.26–1.30)
Model 3, HR (95% CI)	1.00	1.07 (1.01–1.13)	1.09 (1.03–1.15)	1.25 (1.18–1.32)	< 0.001	1.10 (1.08–1.12)
Stroke						
No. of cases/person-years	734/912,415	942/907,589	1049/906,568	1251/906,701	< 0.001	
Model 1, HR (95% CI)	1.00	1.29 (1.17–1.42)	1.44 (1.31–1.58)	1.71 (1.56–1.88)	< 0.001	1.21 (1.17–1.24)
Model 2, HR (95% CI)	1.00	1.05 (0.96–1.16)	1.08 (0.98–1.19)	1.24 (1.13–1.36)	< 0.001	1.08 (1.05–1.12)
Model 3, HR (95% CI)	1.00	0.98 (0.89–1.08)	0.95 (0.86–1.05)	0.99 (0.90–1.10)	0.89	0.98 (0.95–1.02)

TyG, triglyceride-glucose; HR, hazard ratio

Model 1: an unadjusted model

Model 2: adjusted for age, sex, ethnicity (White and others), region (England, Scotland, and Wales), and Townsend Deprivation Index

Model 3: further adjusted for current smoking (yes or no), physical activity (< 150 or  ≥ 150 min/week), body mass index (kg/m^2^), systolic blood pressure (mm Hg), total cholesterol (mg/dL), low-density lipoprotein cholesterol (mg/dL), uric acid (mg/dL), glycated hemoglobin (mmol/mol), estimated glomerular filtration rate (mL/min/1.73 m^2^), high sensitivity C-reactive protein (mg/L), aspirin use (yes or no), insulin treatment (yes or no), antihypertensive medication (yes or no), cholesterol-lowering medication (yes or no), prevalent retinopathy (yes or no), and chronic kidney disease (yes or no)

**Table 3:** Prospective associations between the TG/HDL-C ratio and risk of cardiovascular disease in the UK Biobank

	TG/HDL-C ratio				*P* trend	Per 1 SD increase
	Quartile 1	Quartile 2	Quartile 3	Quartile 4		in Log TG/HDL-C values
Median	1.11	1.90	3.01	5.51		
Cardiovascular disease						
No. of cases/person-years	2817/811,994	4197/807,320	5471/803,410	7269/799,568	< 0.001	
Model 1, HR (95% CI)	1.00	1.50 (1.43–1.57)	1.96 (1.88–2.06)	2.62 (2.51–2.74)	< 0.001	1.40 (1.38–1.42)
Model 2, HR (95% CI)	1.00	1.27 (1.21–1.34)	1.51 (1.44–1.58)	1.91 (1.82–1.99)	< 0.001	1.27 (1.25–1.29)
Model 3, HR (95% CI)	1.00	1.07 (1.02–1.13)	1.13 (1.07–1.18)	1.29 (1.23–1.36)	< 0.001	1.12 (1.10–1.13)
Coronary heart disease						
No. of cases/person-years	2165/814,334	3404/810,261	4569/806,749	6266/803,386	< 0.001	
Model 1, HR (95% CI)	1.00	1.58 (1.50–1.67)	2.13 (2.03–2.24)	2.94 (2.80–3.08)	< 0.001	1.45 (1.43–1.48)
Model 2, HR (95% CI)	1.00	1.34 (1.27–1.41)	1.63 (1.55–1.72)	2.11 (2.01–2.22)	< 0.001	1.32 (1.30–1.34)
Model 3, HR (95% CI)	1.00	1.11 (1.05–1.17)	1.18 (1.11–1.24)	1.37 (1.30–1.45)	< 0.001	1.15 (1.13–1.18)
Stroke						
No. of cases/person-years	739/907,452	919/907,214	1074/907,801	1244/910,805	< 0.001	
Model 1, HR (95% CI)	1.00	1.24 (1.13–1.37)	1.45 (1.32–1.60)	1.68 (1.53–1.84)	< 0.001	1.19 (1.16–1.23)
Model 2, HR (95% CI)	1.00	1.07 (0.97–1.17)	1.14 (1.04–1.26)	1.29 (1.17–1.41)	< 0.001	1.09 (1.05–1.13)
Model 3, HR (95% CI)	1.00	0.97 (0.88–1.08)	0.97 (0.88–1.08)	1.02 (0.92–1.14)	0.34	1.00 (0.96–1.04)

TG/HDL-C, triglyceride to high-density lipoprotein cholesterol; HR, hazard ratio

Model 1: an unadjusted model

Model 2: adjusted for age, sex, ethnicity (White and others), region (England, Scotland, and Wales), and Townsend Deprivation Index

Model 3: further adjusted for current smoking (yes or no), physical activity (< 150 or  ≥ 150 min/week), body mass index (kg/m^2^), systolic blood pressure (mm Hg), total cholesterol (mg/dL), low-density lipoprotein cholesterol (mg/dL), uric acid (mg/dL), glycated hemoglobin (mmol/mol), estimated glomerular filtration rate (mL/min/1.73 m^2^), high sensitivity C-reactive protein (mg/L), aspirin use (yes or no), insulin treatment (yes or no), antihypertensive medication (yes or no), cholesterol-lowering medication (yes or no), prevalent retinopathy (yes or no), and chronic kidney disease (yes or no)

**Fig. 2:**
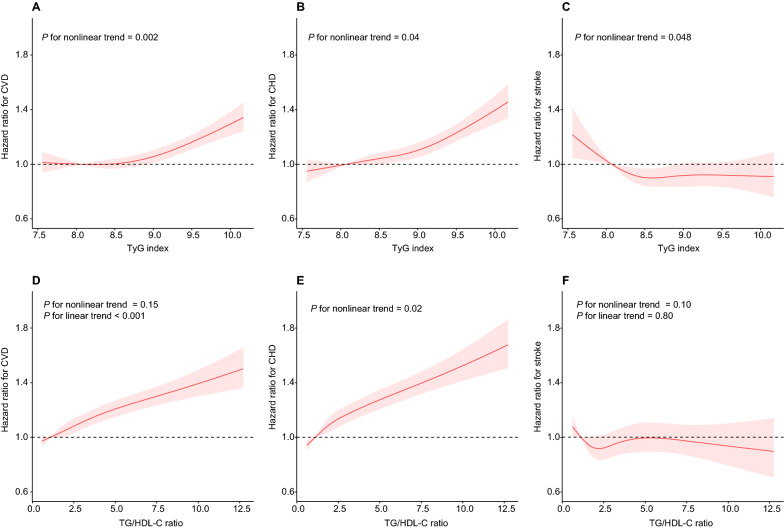
Multivariable-adjusted hazard ratios and 95% confidence intervals of cardiovascular disease associated with the TyG index **A**–**C** and TG/HDL-C ratio **D**–**F**. TyG, triglyceride-glucose; TG/HDL-C, triglyceride to high-density lipoprotein cholesterol. Hazard ratios were adjusted for the same variables included in model 3 in Table 2

Multivariable-adjusted restricted cubic spline analyses suggested a nonlinear association of the TyG index with total CVD, CHD, and stroke. They also indicated a nonlinear association between the TG/HDL-C ratio and CHD. There was no evidence of a nonlinear association of TG/HDL-C ratio with total CVD or stroke. There was evidence of a significant linear relationship of TG/HDL-C ratio with total CVD (*P* for linearity < 0.001), but not with stroke (Fig. 2). In sensitivity analyses additionally adjusted for prevalent dyslipidemia, diabetes, and hypertension (Additional file 1: Table S4) or with exclusion of 6080 incident CVD cases with less than 3 years of follow-up (Additional file 1: Table S5), we still observed significant associations of the TyG index and TG/HDL-C ratio with the risk of total CVD and CHD (all* P* < 0.001).

### Mediation analyses

In mediation analyses (Table 4), dyslipidemia accounted for 45.8% and 36.1% of the associations of the TyG index with incident CVD and CHD, respectively, and 40.0% and 33.5% of the TG/HDL-C ratio’s associations with CVD and CHD, respectively. The proportions mediated through type 2 diabetes were 27.0% and 20.6% for the associations of the TyG index with incident CVD and CHD, respectively, and 11.8% and 9.6% for the TG/HDL-C ratio’s associations with CVD and CHD, respectively. Hypertension also played a significant mediating role in the associations of the TyG index with incident CVD and CHD (proportions mediated: 15.0% and 11.1%, respectively), and in the associations of the TG/HDL-C ratio with incident CVD and CHD (proportions mediated: 13.3% and 10.6%, respectively). The combined mediation effect of dyslipidemia, type 2 diabetes, and hypertension accounted for 56.5% and 43.6% of the total effect of TyG index on CVD and CHD, respectively, and 46.6% and 38.3% of the total effect of TG/HDL-C ratio on CVD and CHD, respectively (All *P* < 0.001, Table 4). There were no significant mediation effects observed for prevalent type 1 diabetes.

**Table 4:** Mediation analysis to evaluate whether prevalent dyslipidemia, type 2 diabetes, and hypertension mediated the associations of the TyG index and TG/HDL-C ratio with cardiovascular disease risk^a^

	Dyslipidemia	*P*	Type 2 diabetes	*P*	Hypertension	*P*	Combined mediation effect	*P*
	Proportion mediated, % (95% CI)		Proportion mediated, % (95% CI)		Proportion mediated, % (95% CI)		Proportion mediated, % (95% CI)	
TyG index^b^								
Cardiovascular disease	45.8 (34.4–57.7)	< 0.001	27.0 (19.5–36.1)	< 0.001	15.0 (9.8–22.2)	< 0.001	56.5 (41.7–70.2)	< 0.001
Coronary heart disease	36.1 (28.0–44.9)	< 0.001	20.6 (15.4–26.9)	< 0.001	11.1 (7.2–16.7)	< 0.001	43.6 (33.6–54.1)	< 0.001
TG/HDL-C ratio^b^								
Cardiovascular disease	40.0 (32.8–47.7)	< 0.001	11.8 (9.1–15.2)	< 0.001	13.3 (9.8–17.8)	< 0.001	46.6 (38.0–55.4)	< 0.001
Coronary heart disease	33.5 (27.9–39.6)	< 0.001	9.6 (7.5–12.3)	< 0.001	10.6 (7.8–14.3)	< 0.001	38.3 (31.8–45.4)	< 0.001

TyG, triglyceride-glucose; TG/HDL-C, triglyceride to high-density lipoprotein cholesterol

^a^Adjusted for age, sex, ethnicity (White and others), region (England, Scotland, and Wales), Townsend Deprivation Index, current smoking (yes or no), physical activity (< 150 or  ≥ 150 min/week), body mass index (kg/m^2^), systolic blood pressure (mm Hg), total cholesterol (mg/dL), low-density lipoprotein cholesterol (mg/dL), uric acid (mg/dL), glycated hemoglobin (mmol/mol), estimated glomerular filtration rate (mL/min/1.73 m^2^), high sensitivity C-reactive protein (mg/L), aspirin use (yes or no), insulin treatment (yes or no), antihypertensive medication (yes or no), cholesterol-lowering medication (yes or no), prevalent retinopathy (yes or no), and chronic kidney disease (yes or no)

^b^Per 1 SD increment in log-transformed values

## Discussion

In this large, prospective, population-based cohort of middle-aged individuals, it was found that elevated baseline TyG index and TG/HDL-C ratio were associated with a higher risk of CVD. The observed associations remained statistically significant after adjustment for the well-established CVD risk factors. Participants in the highest quartile of the TyG index and TG/HDL-C ratio had a 1.19- and 1.29-fold increased risk of total CVD, respectively, compared with those in the lowest quartile. The deleterious associations were largely mediated by the greater prevalence of dyslipidemia, type 2 diabetes, and hypertension. Furthermore, the results were consistent in sensitivity analyses.

Insulin resistance has been shown to promote both atherogenesis and clinically relevant advanced plaque progression and is considered as an important risk factor for CVD [3, 33]. The assessment of insulin resistance requires sophisticated techniques that are unsuitable for large-scale or epidemiological studies [4]. The widespread use of the TyG index and TG/HDL-C ratio as surrogate markers of insulin resistance has attested to their suitability and has generated much evidence that they are associated with the risk of CVD [7–21, 34]. A cross-sectional study revealed a positive relationship between the TyG index and 10-year CVD risk evaluated using the Framingham risk score [8]. Hong et al. [11] performed a retrospective cohort study in a Korean population aged  ≥ 40 and demonstrated that the TyG index was an independent predictor of developing atherosclerotic CVD events during 8.2 years of mean follow-up. Data from a community-based prospective study in the Kailuan cohort suggested that individuals in the highest quartile of the baseline TyG index had a 2.08-fold higher risk of myocardial infarction than those in the lowest quartile [14]. Furthermore, another 8-year prospective study of 796 participants showed that an elevated TG/HDL-C ratio predicted the incident risk of CVD events [21]. Consistent with prior studies, our study of a larger sample size confirmed that higher TyG index and TG/HDL-C ratio were significantly associated with increased risks of total CVD and CHD in the UK Biobank population. Neither the TyG index nor TG/HDL-C ratio was associated with stroke in our population after full covariate adjustment, in contrast with some earlier studies [7, 17], although this association has been inconsistent [19]. We could not rule out the possibility that insufficient power impeded our ability to detect such an association.

Very few studies have explored the association of both the TyG index and TG/HDL-C ratio with CVD risk. A prospective cohort study documented that an elevated TyG index and TG/HDL-C ratio predicted a higher risk and more advanced progression of arterial stiffness in the hypertensive population [35]. In a longitudinal study with a relatively small sample size (n = 732), the multivariable-adjusted hazard ratios for incident CVD were statistically significant when evaluated by the TG/HDL-C ratio, but not by the TyG index [20]. Our results added considerable evidence to the literature of these two indicators on CVD risk, reinforcing their importance as useful and cost-effective early indicators for subclinical atherosclerosis progression and subsequent cardiac and cerebrovascular events.

Another important finding, which to our knowledge has not been reported previously, is the substantial mediating contributions of type 2 diabetes, hypertension, and dyslipidemia on the associations of the TyG index and TG/HDL-C ratio with CVD. Diabetes, hypertension, and dyslipidemia predispose individuals to the accelerated progression of atherosclerosis and CVD [22]. Insulin resistance is a crucial pathophysiological pathway for the development of diabetes, hypertension, and dyslipidemia and is present for an extended period before these manifest diseases are diagnosed [22, 23, 36]. The Vascular-Metabolic CUN cohort suggested that the TyG index was a better predictor than fasting plasma glucose or triglyceride concentrations of future diabetes diagnosis in normoglycemic subjects [37]. In a meta-analysis of eight observational studies, Wang et al. [38] demonstrated that a higher TyG index was independently associated with an increased risk of hypertension in a general adult population. The current study investigated the mediating effects of diabetes, hypertension, and dyslipidemia on the associations of the TyG index and TG/HDL-C ratio with CVD, thereby integrating prior evidence into comprehensive pathways that can be used to guide clinical practice. Our findings reinforced the value of developing effective complex interventions targeting diabetes, hypertension, and dyslipidemia as means to prevent CVD among individuals with insulin resistance.

The strengths of this study included an unprecedented amount of biological and medical data on nearly half a million participants in the UK, prospective study design, complete and long-term follow-up, and comprehensive adjustment for potential cardiovascular risk factors. Moreover, the use of standardized protocols and rigorous quality control procedures for measuring study exposure, mediator and outcome variables contributed to valid evaluation of association and mediation. However, this study also had several limitations. First, a random blood sample was used for biochemistry assays to measure the TyG index and TG/HDL-C ratio in UK Biobank participants, and non-fasting glucose was a major limitation of this study although we adjusted for HbA_1c_ values in multivariable analyses. Second, the TyG index and TG/HDL-C ratio were determined based on a single blood sample at baseline, so we could not assess the effect of their changes on CVD over time. Then, given the observational design of this study, we could not completely exclude residual confounding effects, although we adjusted for several major confounding factors. Finally, the UK Biobank cohort is not nationally representative, suggesting that it may be affected by a “healthy volunteer” selection bias. Nevertheless, valid assessment of associations between exposures and outcomes may be widely generalizable and does not require participants to be representative of the population at large [39].

In conclusion, our analysis of data from the UK Biobank showed that elevated baseline TyG index and TG/HDL-C ratio, two surrogate markers of insulin resistance, were associated with a higher risk of CVD after adjustment for the well-established CVD risk factors. These associations were largely mediated by the greater prevalence of dyslipidemia, diabetes, and hypertension.

## Supplementary Information

**Additional file 1: Table S1.** Characteristics of the study population according to the TG/HDL-C ratio quartiles. TG/HDL-C, triglyceride to high-density lipoprotein cholesterol; eGFR, estimated glomerular filtration rate. aContinuous variables are expressed as mean (SD). Categorical variables are expressed as frequency (percentage). **Table S2.** Correlations of the TyG index and TG/HDL-C ratio with participant characteristicsa. TyG, triglyceride-glucose; TG/HDL-C, triglyceride to high-density lipoprotein cholesterol; eGFR, estimated glomerular filtration rate. aPoint-biserial correlation for dichotomized variables and Pearson’s correlation for continuous variables. **Table S3.** Characteristics and cardiovascular outcomes of the study population in England, Scotland, and Wales. TyG, triglyceride-glucose; TG/HDL-C, triglyceride to high-density lipoprotein cholesterol; eGFR, estimated glomerular filtration rate. aContinuous variables are expressed as mean (SD). Categorical variables are expressed as frequency (percentage). **Table S4.** Sensitivity analysis: Multivariable-adjusted hazard ratios of cardiovascular disease associated with the TyG index and TG/HDL-C ratio, additionally adjusted for prevalent dyslipidemia, type 2 diabetes, and hypertension. TyG, triglyceride-glucose; TG/HDL-C, triglyceride to high-density lipoprotein cholesterol; HR, hazard ratio. aHazard ratios were adjusted for the variables included in model 3 in Table 2 in addition to prevalent type 2 diabetes, hypertension, and dyslipidemia. **Table S5.** Sensitivity analysis: Associations of the TyG index and TG/HDL-C ratio with risk of cardiovascular disease, excluding 6080 incident cases with less than 3 years of follow-up (N = 397,255). TyG, triglyceride-glucose; TG/HDL-C, triglyceride to high-density lipoprotein cholesterol; HR, hazard ratio. Hazard ratios were adjusted for the same variables included in model 3 in Table 2. **Appendix S1.** ICD-10 codes used to ascertain comorbidities and cardiovascular outcomes.

## Data Availability

The dataset supporting the conclusions of this article is available in the public UK Biobank Resource (www.ukbiobank.ac.uk/).
