# Supplementary material for: Triglyceride-glucose index and triglyceride to high-density lipoprotein cholesterol ratio as potential cardiovascular disease risk factors: an analysis of UK biobank data
Source: Cardiovasc Diabetol. 2023 Feb 16;22:34. doi: 10.1186/s12933-023-01762-2 (PMC9936712; doi:10.1186/s12933-023-01762-2)
Supplement: Supplementary file 1 — Additional file 1: Table S1. Characteristics of the study population according to the TG/HDL-C ratio quartiles. TG/HDL-C, triglyceride to high-density lipoprotein cholesterol; eGFR, estimated glomerular filtration rate. aContinuous variables are expressed as mean (SD). Categorical variables are expressed as frequency (percentage). Table S2. Correlations of the TyG index and TG/HDL-C ratio with participant characteristicsa. TyG, triglyceride-glucose; TG/HDL-C, triglyceride to high-density lipoprotein cholesterol; eGFR, estimated glomerular filtration rate. aPoint-biserial correlation for dichotomized variables and Pearson’s correlation for continuous variables. Table S3. Characteristics and cardiovascular outcomes of the study population in England, Scotland, and Wales. TyG, triglyceride-glucose; TG/HDL-C, triglyceride to high-density lipoprotein cholesterol; eGFR, estimated glomerular filtration rate. aContinuous variables are expressed as mean (SD). Categorical variables are expressed as frequency (percentage). Table S4. Sensitivity analysis: Multivariable-adjusted hazard ratios of cardiovascular disease associated with the TyG index and TG/HDL-C ratio, additionally adjusted for prevalent dyslipidemia, type 2 diabetes, and hypertension. TyG, triglyceride-glucose; TG/HDL-C, triglyceride to high-density lipoprotein cholesterol; HR, hazard ratio. aHazard ratios were adjusted for the variables included in model 3 in Table 2 in addition to prevalent type 2 diabetes, hypertension, and dyslipidemia. Table S5. Sensitivity analysis: Associations of the TyG index and TG/HDL-C ratio with risk of cardiovascular disease, excluding 6080 incident cases with less than 3 years of follow-up (N = 397,255). TyG, triglyceride-glucose; TG/HDL-C, triglyceride to high-density lipoprotein cholesterol; HR, hazard ratio. Hazard ratios were adjusted for the same variables included in model 3 in Table 2. Appendix S1. ICD-10 codes used to ascertain comorbidities and cardiovascular outcomes. [file 12933_2023_1762_MOESM1_ESM.docx]

**Triglyceride-glucose index and triglyceride to high-density lipoprotein cholesterol ratio as potential cardiovascular disease risk factors: an analysis of UK Biobank data**

Bizhong Che, Chongke Zhong, Ruijie Zhang, Liyuan Pu, Tian Zhao, Yonghong Zhang, Liyuan Han

**Table S1** Characteristics of the study population according to the TG/HDL-C ratio quartiles

|  | TG/HDL-C ratio | | | | | |
| --- | --- | --- | --- | --- | --- | --- |
| Characteristics^a^ | Total | Quartile 1  < 1.49 | Quartile 2  1.49–2.38 | Quartile 3  2.39–3.89 | Quartile 4  ≥ 3.90 | *P* trend |
| N | 403,335 | 100,838 | 100,866 | 100,796 | 100,835 |  |
| Female | 222,586 (55.2) | 75,845 (75.2) | 62,607 (62.1) | 50,478 (50.1) | 33,656 (33.4) | < 0.001 |
| Age, years | 56.2 (8.1) | 55.1 (8.2) | 56.4 (8.1) | 57.0 (8.0) | 56.5 (8.0) | < 0.001 |
| White ethnicity | 381,880 (94.7) | 95,132 (94.3) | 95,532 (94.7) | 95,686 (94.9) | 95,530 (94.7) | < 0.001 |
| Townsend Deprivation Index | – 1.3 (3.1) | – 1.5 (3.0) | – 1.4 (3.0) | – 1.3 (3.1) | – 1.2 (3.1) | < 0.001 |
| Current smoking | 42,025 (10.4) | 7522 (7.5) | 9497 (9.4) | 10,999 (10.9) | 14,007 (13.9) | < 0.001 |
| Physical activity, min/week |  | | |  |  | < 0.001 |
| < 150 | 184,414 (45.7) | 41,154 (40.8) | 44,698 (44.3) | 47,469 (47.1) | 51,093 (50.7) |  |
| ≥ 150 | 218,921 (54.3) | 59,684 (59.2) | 56,168 (55.7) | 53,327 (52.9) | 49,742 (49.3) |  |
| Body mass index, kg/m^2^ | 27.3 (4.7) | 24.8 (3.8) | 26.7 (4.4) | 28.2 (4.7) | 29.5 (4.7) | < 0.001 |
| Systolic blood pressure, mm Hg | 137.9 (18.6) | 133.8 (19.1) | 137.1 (18.7) | 139.4 (18.2) | 141.3 (17.5) | < 0.001 |
| Diastolic blood pressure, mm Hg | 82.5 (10.1) | 79.8 (10.0) | 81.8 (10.0) | 83.4 (9.9) | 84.8 (9.8) | < 0.001 |
| Uric acid, mg/dL | 5.2 (1.3) | 4.5 (1.1) | 4.9 (1.2) | 5.4 (1.3) | 5.9 (1.3) | < 0.001 |
| Glycated hemoglobin, mmol/mol | 35.9 (6.5) | 34.6 (4.9) | 35.3 (5.3) | 36.2 (6.4) | 37.5 (8.2) | < 0.001 |
| Glucose, mg/dL | 91.9 (21.3) | 89.3 (16.0) | 90.4 (16.8) | 92.1 (20.1) | 95.8 (29.3) | < 0.001 |
| Triglycerides, mg/dL | 153.9 (90.5) | 75.7 (18.0) | 112.6 (22.2) | 157.1 (31.1) | 270.1 (98.3) | < 0.001 |
| Total cholesterol, mg/dL | 222.6 (43.2) | 218.6 (39.8) | 220.6 (41.6) | 223.9 (44.1) | 227.4 (46.5) | < 0.001 |
| High-density lipoprotein cholesterol, mg/dL | 56.4 (14.7) | 71.4 (13.8) | 59.2 (10.5) | 51.8 (9.0) | 43.5 (8.1) | < 0.001 |
| Low-density lipoprotein cholesterol, mg/dL | 139.4 (33.0) | 129.1 (29.4) | 138.3 (31.5) | 144.1 (33.7) | 146.1 (34.4) | < 0.001 |
| High sensitivity C-reactive protein, mg/L | 2.6 (4.3) | 1.8 (3.8) | 2.4 (4.4) | 2.9 (4.5) | 3.2 (4.3) | < 0.001 |
| Creatinine, mg/dL | 0.81 (0.20) | 0.76 (0.15) | 0.80 (0.17) | 0.83 (0.21) | 0.86 (0.25) | < 0.001 |
| eGFR, mL/min/1.73 m^2^ | 91.3 (13.2) | 93.0 (12.4) | 91.2 (12.8) | 90.3 (13.3) | 90.5 (14.0) | < 0.001 |
| Aspirin | 42,153 (10.4) | 7678 (7.6) | 9444 (9.4) | 11,496 (11.4) | 13,535 (13.4) | < 0.001 |
| Insulin | 3702 (0.9) | 966 (1.0) | 703 (0.7) | 827 (0.8) | 1206 (1.2) | < 0.001 |
| Antihypertensive medication | 73,657 (18.3) | 11,375 (11.3) | 16,328 (16.2) | 21,020 (20.8) | 24,934 (24.7) | < 0.001 |
| Cholesterol-lowering medication | 55,459 (13.7) | 8019 (7.9) | 11,722 (11.6) | 15,624 (15.5) | 20,094 (19.9) | < 0.001 |
| Retinopathy | 8868 (2.2) | 1910 (1.9) | 2205 (2.2) | 2318 (2.3) | 2435 (2.4) | < 0.001 |
| Chronic kidney disease | 7956 (2.0) | 1170 (1.2) | 1665 (1.6) | 2284 (2.3) | 2837 (2.8) | < 0.001 |
| Dyslipidemia | 27,032 (6.7) | 3400 (3.4) | 5531 (5.5) | 7632 (7.6) | 10,469 (10.4) | < 0.001 |
| Type 1 diabetes | 1275 (0.3) | 296 (0.3) | 220 (0.2) | 286 (0.3) | 473 (0.5) | < 0.001 |
| Type 2 diabetes | 14,157 (3.5) | 1087 (1.1) | 2101 (2.1) | 3947 (3.9) | 7022 (7.0) | < 0.001 |
| Hypertension | 56,202 (13.9) | 8928 (8.8) | 12,723 (12.6) | 15,823 (15.7) | 18,728 (18.6) | < 0.001 |

TG/HDL-C, triglyceride to high-density lipoprotein cholesterol; eGFR, estimated glomerular filtration rate.

^a^Continuous variables are expressed as mean (SD). Categorical variables are expressed as frequency (percentage).

**Table S2** Correlations of the TyG index and TG/HDL-C ratio with participant characteristics^a^

|  | TyG index |  |  | TG/HDL-C ratio | |
| --- | --- | --- | --- | --- | --- |
|  | *r* | *P* |  | *r* | *P* |
| Male | 0.21 | < 0.001 |  | 0.29 | < 0.001 |
| Age | 0.14 | < 0.001 |  | 0.02 | < 0.001 |
| White ethnicity | –0.03 | < 0.001 |  | 0.003 | 0.10 |
| Townsend Deprivation Index | 0.02 | < 0.001 |  | 0.04 | < 0.001 |
| Current smoking | 0.05 | < 0.001 |  | 0.08 | < 0.001 |
| Physical activity | –0.06 | < 0.001 |  | –0.06 | < 0.001 |
| Body mass index | 0.35 | < 0.001 |  | 0.31 | < 0.001 |
| Systolic blood pressure | 0.21 | < 0.001 |  | 0.12 | < 0.001 |
| Diastolic blood pressure | 0.20 | < 0.001 |  | 0.15 | < 0.001 |
| Uric acid | 0.34 | < 0.001 |  | 0.34 | < 0.001 |
| Glycated hemoglobin | 0.31 | < 0.001 |  | 0.17 | < 0.001 |
| Glucose | 0.40 | < 0.001 |  | 0.13 | < 0.001 |
| Triglycerides | 0.89 | < 0.001 |  | 0.94 | < 0.001 |
| High-density lipoprotein cholesterol | –0.46 | < 0.001 |  | –0.60 | < 0.001 |
| Total cholesterol | 0.22 | < 0.001 |  | 0.07 | < 0.001 |
| Low-density lipoprotein cholesterol | 0.25 | < 0.001 |  | 0.11 | < 0.001 |
| High sensitivity C-reactive protein | 0.11 | < 0.001 |  | 0.09 | < 0.001 |
| Creatinine | 0.12 | < 0.001 |  | 0.15 | < 0.001 |
| eGFR | –0.09 | < 0.001 |  | –0.03 | < 0.001 |
| Aspirin | 0.08 | < 0.001 |  | 0.06 | < 0.001 |
| Insulin | 0.07 | < 0.001 |  | 0.02 | < 0.001 |
| Antihypertensive medication | 0.15 | < 0.001 |  | 0.11 | < 0.001 |
| Cholesterol-lowering medication | 0.16 | < 0.001 |  | 0.11 | < 0.001 |
| Retinopathy | 0.03 | < 0.001 |  | 0.01 | < 0.001 |
| Chronic kidney disease | 0.05 | < 0.001 |  | 0.04 | < 0.001 |
| Dyslipidemia | 0.12 | < 0.001 |  | 0.09 | < 0.001 |
| Type 1 diabetes | 0.05 | < 0.001 |  | 0.02 | < 0.001 |
| Type 2 diabetes | 0.17 | < 0.001 |  | 0.12 | < 0.001 |
| Hypertension | 0.12 | < 0.001 |  | 0.09 | < 0.001 |

TyG, triglyceride-glucose; TG/HDL-C, triglyceride to high-density lipoprotein cholesterol; eGFR, estimated glomerular filtration rate.

^a^Point-biserial correlation for dichotomized variables and Pearson’s correlation for continuous variables.

**Table S3** Characteristics and cardiovascular outcomes of the study population in England, Scotland, and Wales

|  | England | Scotland | Wales | *P* |
| --- | --- | --- | --- | --- |
| Characteristics^a^ |  |  |  |  |
| N | 355,148 | 29,953 | 18,234 |  |
| Female | 195,629 (55.1) | 16,899 (56.4) | 10,058 (55.2) | < 0.001 |
| Age, years | 56.3 (8.1) | 55.9 (8.0) | 55.8 (7.9) | < 0.001 |
| White ethnicity | 334,758 (94.3) | 29,415 (98.2) | 17,707 (97.1) | < 0.001 |
| Townsend Deprivation Index | – 1.4 (3.0) | – 1.1 (3.5) | – 1.8 (2.6) | < 0.001 |
| Current smoking | 36,377 (10.2) | 3640 (12.2) | 2008 (11.0) | < 0.001 |
| Physical activity, min/week |  |  |  | < 0.001 |
| < 150 | 161,355 (45.4) | 14,271 (47.6) | 8788 (48.2) |  |
| ≥ 150 | 193,793 (54.6) | 15,682 (52.4) | 9446 (51.8) |  |
| Body mass index, kg/m^2^ | 27.3 (4.7) | 27.2 (4.7) | 27.9 (4.9) | < 0.001 |
| Systolic blood pressure, mm Hg | 137.7 (18.6) | 139.1 (19.0) | 139.4 (18.6) | < 0.001 |
| Diastolic blood pressure, mm Hg | 82.3 (10.1) | 83.2 (10.2) | 83.8 (10.0) | < 0.001 |
| Uric acid, mg/dL | 5.2 (1.3) | 5.1 (1.3) | 5.2 (1.3) | < 0.001 |
| Glycated hemoglobin, mmol/mol | 35.9 (6.5) | 35.7 (6.3) | 35.9 (6.5) | < 0.001 |
| Glucose, mg/dL | 92.0 (21.2) | 90.5 (21.4) | 90.7 (22.7) | < 0.001 |
| Triglycerides, mg/dL | 153.8 (90.4) | 152.5 (90.5) | 159.7 (92.8) | 0.45 |
| High-density lipoprotein cholesterol, mg/dL | 56.5 (14.8) | 56.6 (14.7) | 55.1 (14.1) | < 0.001 |
| TyG index | 8.7 (0.6) | 8.7 (0.6) | 8.7 (0.6) | < 0.001 |
| TG/HDL-C ratio | 3.1 (2.5) | 3.1 (2.5) | 3.3 (2.6) | 0.15 |
| Total cholesterol, mg/dL | 222.5 (43.2) | 224.7 (43.3) | 221.4 (42.1) | < 0.001 |
| Low-density lipoprotein cholesterol, mg/dL | 139.3 (33.0) | 141.4 (33.2) | 139.3 (32.4) | < 0.001 |
| High sensitivity C-reactive protein, mg/L | 2.5 (4.3) | 2.6 (4.6) | 2.8 (4.6) | < 0.001 |
| Creatinine, mg/dL | 0.81 (0.20) | 0.80 (0.19) | 0.81 (0.19) | < 0.001 |
| eGFR, mL/min/1.73 m^2^ | 91.1 (13.2) | 92.4 (12.9) | 91.5 (13.2) | < 0.001 |
| Aspirin | 36,662 (10.3) | 3408 (11.4) | 2083 (11.4) | < 0.001 |
| Insulin | 3275 (0.9) | 252 (0.8) | 175 (1.0) | 0.31 |
| Antihypertensive medication | 64,942 (18.3) | 5211 (17.4) | 3504 (19.2) | < 0.001 |
| Cholesterol-lowering medication | 49,288 (14.9) | 3435 (11.5) | 2736 (15.0) | < 0.001 |
| Retinopathy | 8214 (2.3) | 356 (1.2) | 298 (1.6) | < 0.001 |
| Chronic kidney disease | 7059 (2.0) | 534 (1.8) | 363 (2.0) | 0.049 |
| Dyslipidemia | 25,306 (7.1) | 468 (1.6) | 1258 (6.9) | < 0.001 |
| Type 1 diabetes | 1170 (0.3) | 62 (0.2) | 43 (0.2) | < 0.001 |
| Type 2 diabetes | 12,953 (3.7) | 563 (1.9) | 641 (3.5) | < 0.001 |
| Hypertension | 51,928 (14.6) | 1834 (6.1) | 2440 (13.3) | < 0.001 |
| Outcomes, No. of cases/person-years |  |  |  |  |
| Cardiovascular disease | 17,608/2,797,572 | 1354/268,452 | 792/156,267 | < 0.001 |
| Coronary heart disease | 14,707/2,808,053 | 1054/269,792 | 643/156,885 | < 0.001 |
| Stroke | 3445/3,159,347 | 341/298,940 | 190/174,984 | 0.01 |

TyG, triglyceride-glucose; TG/HDL-C, triglyceride to high-density lipoprotein cholesterol; eGFR, estimated glomerular filtration rate.

^a^Continuous variables are expressed as mean (SD). Categorical variables are expressed as frequency (percentage).

**Table S4** Sensitivity analysis: Multivariable-adjusted hazard ratios of cardiovascular disease associated with the TyG index and TG/HDL-C ratio, additionally adjusted for prevalent dyslipidemia, diabetes, and hypertension

|  | HR (95% CI)^a^ | | | | | Per 1 SD increment |
| --- | --- | --- | --- | --- | --- | --- |
|  | Quartile 1 | Quartile 2 | Quartile 3 | Quartile 4 | *P* trend | in Log values |
| TyG index |  |  |  |  |  |  |
| Cardiovascular disease | 1.00 | 1.03 (0.98–1.08) | 1.00 (0.96–1.05) | 1.08 (1.03–1.14) | < 0.001 | 1.03 (1.02–1.05) |
| Coronary heart disease | 1.00 | 1.05 (1.00–1.11) | 1.04 (0.98–1.09) | 1.13 (1.07–1.19) | < 0.001 | 1.06 (1.04–1.08) |
| Stroke | 1.00 | 0.97 (0.88–1.07) | 0.92 (0.83–1.02) | 0.92 (0.83–1.02) | 0.09 | 0.96 (0.92–1.00) |
| TG/HDL-C ratio |  |  |  |  |  |  |
| Cardiovascular disease | 1.00 | 1.04 (0.99–1.09) | 1.06 (1.00–1.11) | 1.15 (1.09–1.21) | < 0.001 | 1.06 (1.04–1.08) |
| Coronary heart disease | 1.00 | 1.07 (1.01–1.13) | 1.10 (1.04–1.17) | 1.22 (1.15–1.29) | < 0.001 | 1.09 (1.07–1.11) |
| Stroke | 1.00 | 0.95 (0.86–1.05) | 0.93 (0.84–1.03) | 0.94 (0.84–1.04) | 0.44 | 0.96 (0.93–1.00) |

TyG, triglyceride-glucose; TG/HDL-C, triglyceride to high-density lipoprotein cholesterol; HR, hazard ratio.

^a^Hazard ratios were adjusted for the variables included in model 3 in Table 2 in addition to prevalent diabetes, hypertension, and dyslipidemia.

**Table S5** Sensitivity analysis: Associations of the TyG index and TG/HDL-C ratio with risk of cardiovascular disease, excluding 6080 incident cases with less than 3 years of follow-up (N = 397,255)

|  |  |  |  |  |  | Per 1-SD increment |
| --- | --- | --- | --- | --- | --- | --- |
|  | Quartile 1 | Quartile 2 | Quartile 3 | Quartile 4 | *P* trend | in Log values |
| TyG index |  |  |  |  |  |  |
| Cardiovascular disease |  |  |  |  |  |  |
| No. of cases/person-years | 2093/814,652 | 3057/805,027 | 3700/800,182 | 4824/792,759 | < 0.001 |  |
| HR (95% CI) | 1.00 | 1.07 (1.01–1.13) | 1.08 (1.02–1.14) | 1.21 (1.14–1.28) | < 0.001 | 1.07 (1.05–1.09) |
| Coronary heart disease |  |  |  |  |  |  |
| No. of cases/person-years | 1599/815,921 | 2451/806,602 | 3070/801,827 | 4076/794,696 | < 0.001 |  |
| HR (95% CI) | 1.00 | 1.11 (1.04–1.18) | 1.14 (1.07–1.22) | 1.28 (1.20–1.37) | < 0.001 | 1.10 (1.08–1.13) |
| Stroke |  |  |  |  |  |  |
| No. of cases/person-years | 555/905,684 | 693/897,666 | 769/894,147 | 914/888,358 | < 0.001 |  |
| HR (95% CI) | 1.00 | 0.95 (0.85–1.07) | 0.92 (0.82–1.04) | 0.97 (0.87–1.10) | 0.75 | 0.97 (0.93–1.02) |
| TG/HDL-C ratio |  |  |  |  |  |  |
| Cardiovascular disease |  |  |  |  |  |  |
| No. of cases/person-years | 2005/810,641 | 2941/805,321 | 3811/800,781 | 4917/795,876 | < 0.001 |  |
| HR (95% CI) | 1.00 | 1.07 (1.01–1.14) | 1.13 (1.06–1.19) | 1.27 (1.19–1.35) | < 0.001 | 1.10 (1.08–1.13) |
| Coronary heart disease |  |  |  |  |  |  |
| No. of cases/person-years | 1510/811,904 | 2351/806,870 | 3151/802,466 | 4184/797,806 | < 0.001 |  |
| HR (95% CI) | 1.00 | 1.11 (1.04–1.19) | 1.19 (1.12–1.28) | 1.36 (1.27–1.46) | < 0.001 | 1.14 (1.11–1.16) |
| Stroke |  |  |  |  |  |  |
| No. of cases/person-years | 558/901,479 | 680/897,641 | 790/894,827 | 903/891,908 | < 0.001 |  |
| HR (95% CI) | 1.00 | 0.96 (0.85–1.08) | 0.96 (0.85–1.08) | 1.00 (0.89–1.14) | 0.54 | 0.98 (0.94–1.03) |

TyG, triglyceride-glucose; TG/HDL-C, triglyceride to high-density lipoprotein cholesterol; HR, hazard ratio.

Hazard ratios were adjusted for the same variables included in model 3 in Table 2.

**Online Appendix** ICD-10 codes used to ascertain comorbidities and cardiovascular outcomes

| Dyslipidemia | Type 1 diabetes | Type 2 diabetes | Hypertension |
| --- | --- | --- | --- |
| E78 Disorders of lipoprotein metabolism and other lipidaemias | E10 Insulin-dependent diabetes mellitus | E11 Non-insulin-dependent diabetes mellitus | I10 Essential (primary) hypertension  I11 Hypertensive heart disease  I12 Hypertensive renal disease  I13 Hypertensive heart and renal disease  I15 Secondary hypertension |
| Retinopathy | | Coronary heart disease | Stroke |
| E10.3 Insulin-dependent diabetes mellitus with ophthalmic | | I20 Angina pectoris | I60 Subarachnoid haemorrhage |
| complications  E11.3 Non-insulin-dependent diabetes mellitus with ophthalmic complications  E12.3 Malnutrition-related diabetes mellitus with ophthalmic complications  E13.3 Other specified diabetes mellitus with ophthalmic complications  E14.3 Unspecified diabetes mellitus with ophthalmic complications  H28.0 Diabetic cataract  H33 Retinal detachments and breaks  H35.3 Degeneration of macula and posterior pole  H36.0 Diabetic retinopathy | | I21 Acute myocardial infarction  I22 Subsequent myocardial infarction  I23 Certain current complications following acute myocardial infarction  I24 Other acute ischaemic heart diseases  I25 Chronic ischaemic heart disease | I61 Intracerebral haemorrhage  I62 Other nontraumatic intracranial haemorrhage  I63 Cerebral infarction  I64 Stroke, not specified as haemorrhage or infarction |
| H40-H42 Glaucoma |  |  |  |
|  |  |  |  |
